# Benefits of aromatase blockers for increased testosterone in poultry: A mini-review

**DOI:** 10.14202/vetworld.2025.1190-1198

**Published:** 2025-05-17

**Authors:** Rizki Fitrawan Yuneldi, Claude Mona Airin, Sarmin Sarmin, Andhika Yudha Prawira, Pudji Astuti

**Affiliations:** 1 Research Center for Applied Zoology, Research Organization for Life Science and Environment, National Research and Innovation Agency (BRIN), Cibinong, Indonesia; 2 Department of Physiology, Faculty of Veterinary Medicine, Universitas Gadjah Mada, Yogyakarta, Indonesia

**Keywords:** endocrine modulation, natural aromatase blockers, performance enhancement, poultry reproduction, synthetic aromatase blockers, testosterone

## Abstract

Testosterone is a key androgenic hormone in male poultry, regulating growth performance, reproductive function, and the development of secondary sexual characteristics. However, endogenous testosterone levels are often diminished through conversion to estrogen through the aromatase enzyme, presenting a physiological constraint in poultry production systems. While synthetic testosterone administration has been employed to overcome this limitation, it is frequently accompanied by adverse effects, including gonadal atrophy and impaired spermatogenesis. Consequently, aromatase blockers have emerged as a promising strategy to enhance testosterone levels by inhibiting estrogen biosynthesis. This review synthesizes current evidence on both synthetic aromatase blockers (SABs), such as letrozole and tamoxifen, and natural aromatase blockers (NAB), including compounds derived from *Anadara granosa* and *Anadara nodifera* clamshells, plant extracts, and trace minerals like zinc. The mechanisms, efficacy, physiological effects, and safety profiles of NAB are comparatively examined against SAB. The findings indicate that NAB can significantly elevate testosterone levels and improve reproductive and performance traits without the adverse histopathological effects observed with prolonged SAB or synthetic androgen use. This review highlights the potential of NAB as sustainable alternatives to synthetic hormones in poultry production and recommends further investigations to optimize dosing regimens, elucidate long-term effects, and explore combinatorial strategies.

## INTRODUCTION

Testosterone is a critical androgen that regulates spermatogenesis, sexual function, and metabolic processes in male poultry [1–5]. The hormone also plays a central role in governing performance traits in roosters, including growth and reproductive efficacy [6]. However, endogenous testosterone is readily converted into estradiol through the enzymatic action of aromatase, thus limiting the persistence of high circulating testosterone levels [7]. To address this physiological limitation, synthetic testosterone has been administered to male poultry to enhance growth and reproductive performance [1, 2, 8]. Nevertheless, repeated administration of exogenous testosterone induces negative feedback mechanisms, resulting in the downregulation of endogenous testosterone production over time [9]. Such hormonal interventions are further associated with adverse effects, including testicular atrophy, structural alterations in the seminiferous tubules, and overall reproductive dysfunction [10–13].

Younis *et al*. [10] reported that although synthetic testosterone at 0.1 mg/kg body weight (BW) can improve performance in broiler chickens. It can adversely affect gonadal histology in both sexes [11]. Yuneldi *et al*. [12] demonstrated that the use of synthetic testosterone in *Pelung* chickens leads to testicular atrophy and degeneration of seminiferous tubules, while administration in layer chickens result in reduced testicular mass [2]. Continuous use has been implicated in male infertility [13]. These findings underscore the pressing need for alternative strategies to elevate testosterone levels safely and effectively.

Aromatase blockers offer a promising solution by inhibiting the activity of the aromatase enzyme, thereby reducing estrogen synthesis and consequently increasing circulating testosterone concentrations [14–18]. Aromatase, encoded by the *CYP19A1* gene and localized in the endoplasmic reticulum (ER), is expressed in several tissues, including the adrenal glands, gonads, adipose tissue, and brain [19–27]. Aromatase inhibitors are classified into synthetic and natural categories. Synthetic aromatase blockers (SAB) - such as fadrozole, anastrozole, letrozole, exemestane, and tamoxifen - have been extensively used and studied in poultry production systems [11, 28]. In contrast, natural aromatase blockers (NAB) - including *Anadara granosa* and *Anadara nodifera* clamshell powders, as well as extracts from broccoli, cauliflower stems, mushrooms, and other plants - have received comparatively limited scientific attention [1–3, 12, 29–34].

Despite growing interest in NABs as a safer alternative, several research gaps persist. First, most existing studies have focused predominantly on SABs, while comprehensive evaluations of the physiological efficacy, mechanisms of action, and safety of NABs in poultry remain scarce. Second, the comparative effects of NABs on testosterone production, reproductive outcomes, and secondary sex characteristics are yet to be fully elucidated. Third, long-term data on the impact of NABs on hormonal regulation and poultry performance are lacking, thereby limiting their adoption in commercial settings. In addition, the interaction of NABs with other feed components and their cost-effectiveness compared to SABs remain unexplored.

Therefore, the aim of this mini-review is to systematically examine the existing literature on natural and SABs, with a particular emphasis on natural compounds. This study seeks to evaluate the comparative efficacy of NABs in enhancing testosterone levels and improving performance traits in male poultry, identify potential risks and benefits, and propose future research directions to bridge the knowledge gaps. By compiling available data on both synthetic and natural interventions, this review provides a comprehensive framework for advancing the use of NABs as a sustainable and safe alternative to synthetic testosterone in poultry production.

## NABS AND THEIR EFFECT ON TESTOSTERONE LEVELS IN POULTRY

NABs have been shown to effectively elevate testosterone concentrations in poultry. Among the most promising NABs are clamshell powders derived from *A. granosa* and *A. nodifera*, which contain essential microminerals such as Zn, Mg, Ca, Na, Fe, and K [30]. Zn functions as a potent aromatase inhibitor. These substances have demonstrated consistent and significant increases in testosterone levels across multiple avian species [1, 2, 6, 30]. For instance, in day-old-chick (DOC) layer hens, daily oral administration of 0.036 mg/40 g BW of clamshell powder for 35 days increased testosterone concentrations from 0.019 ng/mL (control) to 0.540 ng/mL [2]. Likewise, in male canaries, a daily dose of 0.3 mg/30 g BW over 21 days elevated testosterone levels from 0.035 ng/mL to 0.305 ng/mL [1]. In *Pelung* chickens, a 56-day treatment with 0.9 mg/kg BW of *A. granosa* clamshell powder increased testosterone levels from 0.13 ng/mL to 1.42 ng/mL [6].

In Bangkok chickens, administration of *A. nodifera* clamshell powder at 6.6 g/day for 35 days raised testosterone levels to 0.23 ng/mL, compared to 0.13 ng/mL in controls. A combined formulation of 6.6 g/day *A. nodifera* and 3.3 g/day milkfish thorn powder further elevated levels to 0.44 ng/mL [31]. These findings demonstrate the capacity of NABs – particularly those derived from *A. granosa* and *A. nodifera* – to inhibit aromatase activity, thereby preventing the conversion of testosterone to estradiol and enhancing androgenic hormone retention.

## SABS AND THEIR INFLUENCE ON TESTOSTERONE LEVELS IN POULTRY

SABs function by directly inhibiting the aromatase enzyme, thereby increasing testosterone concentrations. As noted by Korani [35], aromatase inhibitors in general lead to elevated testosterone levels. In roosters, administration of exemestane at a dose of 0.5 mg/roosters for 60 days raised testosterone levels to 4.24 nmol/L compared with 3.27 nmol/L in controls, accompanied by a notable reduction in aromatase activity [36]. Letrozole at a dose of 0.5 mg/bird/day similarly increased testosterone levels to 5.23 ng/mL, compared with 4.95 ng/mL in the control group [22]. Tamoxifen at 10 mg/kg BW also led to elevated testosterone in broiler chickens [11]. Luo *et al*. [37] observed that administration of letrozole (0.25 mg/kg BW) to 56-week-old roosters improved testosterone levels relative to untreated controls.

Although SABs have demonstrated effectiveness, potential adverse effects should be considered. Younis *et al*. [11] reported that tamoxifen administration caused histological changes in the testes and ovaries of both male and female broiler chickens, affecting their typical structure. These outcomes are summarized in Table 1 [1, 2, 6, 11, 12, 22, 36–38].

**Table 1:** Summary of the effects of NAB, combined therapy, and SAB on testosterone levels, reproductive function, other effects, and side effects in poultry.

NAB/SAB	Poultry and dose	Testosterone levels		Treatment time	Testosterone levels	Reproductive function and other effects	Side effects	References

		NAB^a^/Combined NAB + Fishbone^b^/SAB^c^ (ng/mL)	Control (ng/mL)					
*A. nodifera* clamshell powder	Male Canary 0.3 mg/30 g BW	0.035^a^*	0.305	21 day	Increase	The expression of the CYP19 aromatase receptor was depressed in the syrinx, brain, and testis.	No side effects	[1]
*A. granosa* clamshell powder	Layer chicken 0.036 mg/40 g BW	0.540^a^*	0.019	35 day	Increase	Increase in testes weight	No side effects	[2]
*A. granosa* clamshell powder	Pelung chicken 0.9 mg/kg BW	1.42^a^*	0.13	56 day	Increase	Improved frequency of crowing, growth performance, and pectoralis muscle, especially the chest circumference, fascicle area, number of myofiber in one fascicle, myofiber area, and PCNA-positive cells.	No side effects	[16, 22]
Tamoxifen	Cobb Avian48 chicks (Broiler) 10 mg/kg BW	-^c^	-	3^rd^–9^th^ day (5 and 6 weeks slaughtering age)	Increase	Increased broiler chicken performance	Side effects on testicular and ovarian histology of male and female broiler chickens, affecting the typical structures.	[11]
Letrozole	Post-peak Ross 308 Roosters (age 40 week) 0.5 Letrozole/bird/day	5.23^c^*	4.95	12 week	Increase	Increased total motility (%), forward motility (%), plasma membrane integrity (%), ejaculate volume (mL), sperm concentration (×10^9^), and lower estradiol levels compared with controls	No side effects.	[22]
Exemestane	Rooster 0.5 mg/roosters	4.24 nmol/L^c^*	3.27 nmol/L	60 day	Increase	Increased semen parameters (Concentration 1×10^9^/mL, Total sperm motility %, Progressive motility %, Viability %, Membrane integrity %), lower aromatase enzyme activity, and mitochondrial activity.	No side effects.	[36]
Letrozole	Roosters aged (Age 56 weeks) 0.25 mg/kg BW	>700 µg/mL^c^*	>600 µg/mL	42 day	Increase	Up-regulate the expression of genes related to steroid hormone synthesis, cell differentiation and proliferation, and electron transfer activity, enhance mitochondrial activity, increase testicular weight, and ultimately improve the semen quality of aged roosters. Furthermore, 8 genes including STAR, CYP17A1, NSDHL, SULT1E1, EHF, NRNPA1, PLIN2, and SDHA, were identified as key letrozole genes that regulate semen quality in aged roosters.	No side effects.	[37]
*A. nodifera* clamshell powder	Bangkok chicken 6.6 g BW	0.23^a^	0.13	35 day	Increased but not significant	Clamshells (NAB) and fish thorns boosted muscle performance (springiness, total number and area of myofibers, and PCNA immunoreactivity are present), as well as increased testosterone levels in pectoralis muscle.	No side effects	[38]
*A. nodifera* clamshell powder	Bangkok chicken 6.6 g/day NAB + 3.3 g/day fishbone	0.44^b^*	0.13	35 day	Increase			[38]

Description:

* =Significant, -=Closed access article (Abstract: No numerical data on testosterone levels). NAB=Natural aromatase blockers, SAB=Synthetic aromatase blockers, BW=Body weight, *A. nodifera=Anadara nodifera, A.granosa=Anadara granosa.*

## ADDITIONAL BENEFITS OF AROMATASE BLOCKERS IN POULTRY

Beyond their ability to increase testosterone levels, aromatase blockers – particularly NABs – offer additional physiological benefits. Rosati *et al*. [39] found that aromatase P450 (CYP19) is undetectable in the testes of male songbirds, suggesting that neuroactive estrogen is synthesized in the brain from circulating androgens to mediate male-specific behaviors. In canaries, Astuti *et al*. [1] demonstrated that *A. nodifera* clamshell powder reduced CYP19 expression in the brain, syrinx, and testes. Consequently, vocal duration increased significantly from <1 s to 17 s following NAB treatment at 0.3 mg/30 g BW [1, 40]. Similarly, Yuneldi *et al*. [6] reported increased crowing frequency in *Pelung* chickens after administration of 0.9 mg/kg BW *A. granosa* clamshell powder. NABs have also been associated with enhanced pectoralis muscle development and overall performance in *Pelung* chickens [12].

However, some effects may be limited. For example, administration of *A. granosa* clamshell powder at 0.036 mg/40 g BW did not significantly alter comb size in DOC layer males [41]. In Bangkok chickens, treatment with *A. nodifera* powder at 6.6 g/day for 35 days increased testicular weight without impairing spermatogenesis [42]. When combined with milkfish thorn powder (3.3 g/day), testosterone levels in the pectoralis muscle were enhanced (NAB alone: 1.55 ng/100 g; combination: 1.61 ng/100 g; control: 1.46 ng/100 g) [31, 38, 43, 44]. These regimens also improved metabolic efficiency, thyroid hormone responses, comb morphology, and muscle growth.

Other plant-based NABs, such as mushroom and nettle extracts, have also demonstrated potential in improving performance and inducing sex reversal in broiler chickens [33]. *In ovo* injection of garlic and tomato extracts (0.1 mg/egg) promoted female-to-male sex reversal and supported embryonic development [45]. Green tea extract (0.1 mL/egg) administered on day 5 of incubation led to 80% sex reversal and significantly enhanced feed intake and weight gain during rearing [46]. These effects suggest that NABs may modulate early embryonic sex differentiation through hormonal pathways, offering viable alternatives to SABs without adverse impacts on growth or reproduction [47].

In terms of SAB benefits, Bazyar *et al*. [36] demonstrated that exemestane improved semen quality in aging broiler breeder roosters by enhancing sperm concentration, motility, viability, and mitochondrial function, despite no changes in semen volume or morphology. Letrozole treatment led to enlarged seminiferous tubules and increased sperm count in both the seminiferous epithelium and epididymis [39, 48, 49]. Tamoxifen (10 and 20 mg/kg BW) improved growth performance and carcass yield in broilers without altering sex hormone profiles [50]. Fadrozole hydrochloride injected *in ovo* (0.1 mL) induced 100% sex reversal and increased feed intake and weight gain during the growth period [46]. Notably, excessive aromatase activity in roosters may compromise reproductive performance, while its function in quail appears to synergize with androgens to support spermatogenesis during the breeding season [39, 51].

## MECHANISMS BY WHICH NATURAL AND SABS INCREASE TESTOSTERONE LEVELS

The primary mechanism of aromatase blockers - both natural and synthetic - involves inhibition of the aromatase enzyme, thereby blocking the conversion of testosterone to estradiol. This review provides an updated mechanistic model, particularly for NABs, emphasizing the role of zinc (Zn) as a key micronutrient in testosterone biosynthesis.

Clamshell powders from *A. granosa* and *A. nodifera* contain essential microminerals such as Zn, Mg, Ca, Na, Fe, and K [30]. Zn functions as a potent aromatase inhibitor. According to Yuneldi *et al*. [12], Zn’s action occurs primarily in the ER. Here, Zn^²+^ acts as a second messenger, activating casein kinase 2 (CK2), which phosphorylates aromatase and inhibits its enzymatic activity, thus preserving testosterone levels.

**Figure 1:**
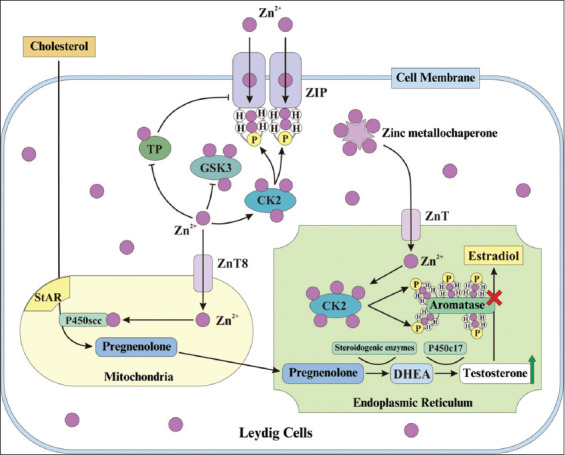
Summary illustration of the mechanism of action of Zn as a natural aromatase blockers in improving testosterone levels [1, 6, 12, 30, 52–62]. Description: ZIP=Zirt and irt-like protein, CK2=Casein kinase 2, GSK3=Glycogen synthase kinase 3, TP=Tyrosine phosphatase, ZnT8=Zn transporter 8, StAR=Steroidogenic acute regulatory protein, DHEA=Dehydroepiandrosterone.

The proposed mechanism, depicted in Figure 1 [1, 6, 12, 30, 52–62], unfolds as follows: clamshell powder is digested, and Zn^²+^ is absorbed in the intestine and enters circulation [30, 52]. It is then transported into Leydig cells through Zrt- and Irt-like protein (ZIP) transporters [52–57]. Within the cytoplasm, Zn^²+^ activates CK2 and inhibits glycogen synthase kinase 3 (GSK3), enhancing Zn uptake through feedback mechanisms [55, 56, 58]. Elevated Zn^²+^ also inhibits tyrosine phosphatase (TP), promoting ZIP phosphorylation and increased Zn^²+^ transport [56]. Zn^²+^ enters mitochondria through Zn transporter 8 (ZnT8), where it facilitates the action of steroidogenic acute regulatory protein (StAR), aiding cholesterol import into mitochondria [59]. There, Zn^²+^ supports the conversion of cholesterol to pregnenolone through P450scc [57, 60], which is further processed into dehydroepiandrosterone (DHEA) and ultimately testosterone through P450c17 in the ER [60].

Zn metallochaperones regulate Zn^²+^ distribution from ZIP to ZnT to maintain cytosolic balance [56, 57, 61]. Simultaneously, Zn^²+^ phosphorylation of aromatase in the ER further suppresses its activity [6, 30, 57, 61]. While this outlines one pathway, other signaling mechanisms also exist. For instance, ZnT8 plays a role in testosterone production through the protein kinase A (PKA) pathway; disruption of ZnT8 expression impairs testosterone synthesis [57, 63].

SABs function through different mechanisms. Exemestane mimics androstenedione to inhibit aromatase activity [20], tamoxifen blocks estrogen receptors to reduce estrogenic signaling [64, 65], and letrozole binds to the heme iron of cytochrome P450 enzymes to deactivate aromatase enzymatic function [20, 66, 67].

## CONCLUSION

This review consolidates current knowledge on the role of aromatase blockers – both natural and synthetic - in enhancing testosterone production and improving reproductive and performance traits in poultry. The evidence presented demonstrates that while SABs, such as exemestane, letrozole, and tamoxifen, effectively increase testosterone levels, their repeated or high-dose use is often associated with adverse effects, including gonadal histopathology and hormonal imbalances. In contrast, NABs, particularly those derived from *Anadara granosa* and *A. nodifera* clamshell powders, as well as plant-based extracts (e.g., green tea, garlic, mushroom), show promising potential to elevate testosterone levels safely, improve muscle performance, influence vocal behaviors, and even facilitate sex reversal during embryogenesis without detectable negative side effects.

The primary strength of this review lies in its comprehensive synthesis of existing literature comparing NABs and SABs in poultry species, along with a mechanistic model detailing the role of trace minerals such as Zn in testosterone biosynthesis. This integrated perspective provides a clearer understanding of the physiological basis and practical implications of aromatase inhibition strategies in poultry husbandry.

However, this review also acknowledges several limitations. The available data on NABs are limited in scope, often restricted to a few specific sources (e.g., clamshell powder) and poultry species. The absence of large-scale, long-term, and comparative trials limits the generalizability of these findings. Moreover, variations in experimental design, dosing regimens, and hormonal assessment techniques across studies pose challenges for standardizing conclusions.

Future research should prioritize well-designed, controlled studies to evaluate the long-term efficacy, optimal dosage, safety, and economic feasibility of NABs across diverse poultry breeds and production systems. Investigations into molecular pathways, potential synergistic effects with other natural compounds, and the impact on offspring development and productivity are warranted. Expanding the exploration of underutilized natural sources, including those with ethnoveterinary relevance, may yield novel candidates for aromatase inhibition. Furthermore, integrative approaches combining nutrigenomics, endocrinology, and animal performance metrics will be instrumental in advancing the application of NABs as sustainable, safe alternatives to synthetic hormones in poultry farming.

In conclusion, NABs represent a promising frontier in poultry endocrinology and reproductive management. With further validation and optimization, NABs could serve as effective, low-risk alternatives for enhancing productivity while maintaining animal welfare and food safety standards.

## AUTHORS’ CONTRIBUTIONS

PA, RFY, CMA, and SS: Planned and designed the review. PA and RFY: Drafted the manuscript. SS and CMA: Literature review and revised the manuscript. PA, RFY, CMA, SS, and AYP: Proofread and revised the manuscript. All authors have read and approved the final manuscript.
